# The role of radiologist in the changing world of healthcare: a White Paper of the European Society of Radiology (ESR)

**DOI:** 10.1186/s13244-022-01241-4

**Published:** 2022-06-04

**Authors:** European Society of Radiology (ESR), Adrian P. Brady, Regina G. Beets-Tan, Boris Brkljačić, Carlo Catalano, Andrea Rockall, Michael Fuchsjäger

**Affiliations:** European Society of Radiology (ESR), Am Gestade 1, 1010 Vienna, Austria

**Keywords:** Radiology, Radiologists, Role of radiologists, Education and training, Healthcare

## Abstract

Radiology as a specialty has been enormously successful since its beginnings, moving over time from an adjunct to clinical decision-making to a crucial component of multidisciplinary patient care. However, this increased centrality of radiology and reliance on our services carries within it dangers, prominent among them being the danger of our being viewed as deliverers of a commodity, and the risk of our becoming overwhelmed by increasing workload, unable to interact sufficiently with patients and referrers due to pressure of work. With this White Paper, the Board of Directors of the European Society of Radiology (ESR) seeks to briefly explain the position of the radiologist in the modern healthcare environment, considering our duties and contributions as doctors, protectors, communicators, innovators, scientists and teachers. This statement is intended to serve as a summary of the breadth of our responsibilities and roles, and to assist radiologists in countering misunderstanding of who we are and what we do.

## Key points

- The role of the radiologist in the modern, rapidly changing world of healthcare is multifaceted and essential.
- Radiologists play a key part in the diagnosis, treatment, and protection of patients.
- Through research and application of novel technologies, radiologists contribute heavily to medical innovation.
- The future of radiology, and healthcare in general, relies upon the quality of next generation radiologists and the ability to engage and work with other specialties.
- Viewpoints on radiology that consider it as a commodity are incorrect, and ignore much of the activity, relevance and value-creation of modern radiologists.

Radiologists were not there at the beginning of radiology. Neither were radiation oncologists or nuclear medicine physicians. The use of X-rays for diagnosis and therapy was pioneered by interested individuals who could see the potential value of the newly-discovered type of ionising radiation, often physicians, surgeons and physicists [1]. It took some decades for radiology and radiotherapy (and later, nuclear medicine) to establish themselves as independent specialties, separate from other disciplines, and responsible for the growing contributions of ionising radiation (and other allied modalities) to healthcare. Initially, radiology was a single field, with any competent radiologist expected to be familiar with all its applications. As knowledge and capabilities grew, subspecialties began to emerge within radiology; the entire field became too broad for any one individual to master, and the benefits of high-level knowledge and service delivery by doctors working exclusively in their particular subspecialty became clear.

Over the past 30 years, the centrality of radiology in patient care, and the impact (immediate and long-term) of radiology in healthcare, have both grown substantially, driven by several factors. Two of the most important are digitisation of information and speed of acquisition [crudely epitomised by picture archiving and communication system (PACS) and spiral computed tomography (CT)]. In the pre-PACS era, image acquisition and reporting were relatively slow analogue processes; it was not uncommon for days to pass between a patient having a CT performed and the report for that study being transcribed and transmitted to the referrer [2]. Greater reliance on clinical examination and history-taking was necessary for diagnosis than is the case nowadays. In the era of single-slice CT, the power of the technique was limited. A body CT was inevitably a sampling process, often with inter-slice gaps, and with limited resolution. The period from the 1990s onwards removed these limitations on rapid information-transfer and cross-sectional imaging capability. PACS and voice-recognition reporting resulted in study reports often being available across the enterprise within minutes of completion of the study. Rapid multi-slice CT [and other developments, not least the emerging power of magnetic resonance imaging (MRI)], resulted in huge improvements in spatial and temporal resolution of cross-sectional imaging. These developments ushered in a “golden age” of radiology participation in patient care: multi-disciplinary decision-making became the standard of care, with radiology at the heart of team-based activity.

So, with increasing power of our tools and skills, and increased reliance on our contributions, is the future bright for our specialty? Not necessarily. In some ways, we have fallen victim to our own success. Certainly, in many ways, imaging has replaced much clinical triage and assessment; imaging is often now requested, performed, and interpreted in acute settings before patients are fully assessed clinically, and imaging reports now frequently supplant clinical judgements. Heavy reliance is placed on our opinions, but this has resulted in often-unsustainable demand for increasing numbers of studies and immediate reports. We are in danger of becoming faceless purveyors of large numbers of outputs (reports), tied to workstations which continually bombard us with more work, hidden away from our referring colleagues and patients, reporting machines with a pulse [3, 4]. Our work contribution is often measured by the volume of work done, the number of studies reported [5, 6], rather than by the impact of that work [7–10]. These trends are unhealthy and dangerous for our patients and our profession. Given these demands, it is hardly surprising that a recent Medscape survey found that 47% of radiologists are suffering from burnout [11]. With this in mind, the Board of Directors of the European Society of Radiology (ESR) wishes, with this paper, to summarise the appropriate role of the radiologist in the modern, rapidly-changing world of healthcare.

## The radiologist as doctor

Radiologists are clinical doctors, trained in our specialty just like any other specialist, with specific expertise that does not imply limitations on our clinical knowledge and value. It seems strange to have to make (and continually emphasise) this central point, but increasing demand for imaging can lead to radiologists being overwhelmed by siloed work, with a resulting danger of loss of visibility and awareness by others of our clinical knowledge and skills.

Our primary clinical roles are in diagnosis and treatment of patients. Interventional radiology is a subspecialty devoted to active in-person treatment delivery and thus interventional radiologists are visible to clinical colleagues and patients. Such visibility is less automatic for radiologists whose work is predominantly diagnostic. As radiology’s capabilities have increased, treatment decisions have increasingly come to depend on imaging findings. The role of the radiologist has gradually shifted from remotely reporting imaging findings to being a contributor to multidisciplinary management teams equal to the other members, specifically at the start of any therapeutic journey, allowing radiologists take on a more central role in clinics, with greater visibility to referring colleagues and patients [12–14]. This shift requires radiologists to be fully knowledgeable about the diseases we deal with, the relevant clinical questions and the impact of imaging findings on treatment choice and outcome. Radiologists therefore devote time and attention to gaining understanding of developments in disease concepts and treatment options, with enhanced communication with referring colleagues to maintain the currency of our knowledge in medical fields outside radiology. By combining our skills in imaging with understanding of clinical management of disease, we radiologists add significant value to patient care [7–9]. Furthermore, we contribute to preventing the development of clinical disease; many screening programmes (e.g., breast cancer, colon cancer, lung cancer) rely heavily on imaging-based determination of the presence or absence of pre-clinical disease, and radiologists are central contributors to these programmes.

These, and many other contributions of value in healthcare by radiologists, are discussed in greater depth in a number of ESR publications exploring the growing value-based radiology movement [7–10, 15].

## The radiologist as protector

Radiologists and allied staff have a key role in ensuring and enhancing patient safety [16]. Fundamental to this is protection of patients from excessive, unjustified or inappropriate exposure to ionising radiation. It is vital that patient exposure to medical radiation be always carefully thought-out following the appropriate indication, and only done when such exposure represents the best means of obtaining necessary information. Alternative non-radiation-based imaging modalities (or other investigations carrying no risk of harm) should always be considered. Radiologists are central to this protective function, based on our broad knowledge of imaging modalities’ capabilities, safety and limitations [17]. When use of ionising radiation is justified, limitation of the dose delivered depends on close cooperation between radiologists and radiographers, and on use of tools such as dose reference levels and optimised protocols [16]. Choice of imaging studies should be informed by standards, guidelines and education of referrers; much of this can be achieved by the incorporation into study requesting chains of decision support tools, such as the ESR iGuide [17].

There are many other aspects of patient safety, which are the ultimate responsibility of radiologists and radiographers, including matters relating to optimisation of imaging, contrast usage, infection prevention, patient handling, informed consent, communication of results, etc. [16].

Patients are becoming increasingly empowered to play an active role in their care, replacing older notions of paternalism and passivity; this is a very appropriate and welcome development. As radiologists, we act as advocates, conduits for information and direct providers of care to patients. We make the necessary efforts to understand patients’ needs and wishes in designing and delivering our services [18].

The radiologist of the twenty first century also has a responsibility to consider environmental protection in the course of their work. Imaging machines, PACS workstations and disposable equipment all carry a cost in carbon and waste. We have an important societal role in measuring and reducing these impacts whilst maintaining patient care. Radiologists need to urgently implement and promote every possible means to work sustainably [19–21].

## The radiologist as communicator

The role of the radiologist as communicator is one that is often under-valued or under-emphasised, yet it is one of the most vital links in the chain of our involvement in patient care. The work of the diagnostic radiologist can, to some extent, be summarised as the sifting of relevant information from the large amount of data presented by imaging studies, the interpretation of that information to identify clinically-relevant findings, the synthesis of a diagnostic and prognostic report from those findings, and ultimately the communication of that report, and its clinical meaning, to the referrer (and, increasingly, directly to the patient) [2, 18]. Radiologists are not there to simply generate reports; those reports must be clear, unambiguous (where possible), structured and communicated efficiently to their recipients. Understanding and acceptance of a radiology report by a referrer is enhanced by close working relationships, which can be lost if the report is treated as a commodity, regardless of its source (especially if there is over-reliance on teleradiological reporting) [22]. We devote time and attention to training our future colleagues in communication skills, and must be supported with time and resources to fulfil this communicative function in our work [6]. We ensure that our reports are not simply lists of findings, regardless of relevance or importance, but rather are actionable and practical in contributing positively to patient care and management [23]. Ring-fencing of time for preparation and conduct of multi-disciplinary team activity is essential to ensure clear communication of often-complex information [6]. Provision of tools for rapid, traceable and verifiable communication of unexpected, urgent or critical findings to referrers should be intrinsic to any well-designed radiology reporting system.

Direct communication with patients is, quite appropriately, increasingly being sought by those to whom our services are delivered [18]. Past models of care, whereby imaging findings were communicated between the radiologist and referrer, and management decisions were arrived at and acted upon, while the patient was a passive participant in the decision-making process, are no longer acceptable to most patients, nor are they appropriate. Patient-centred care implies patient involvement in all major decisions; such involvement is only meaningful if patients are fully-briefed on relevant information, including radiology findings. As radiologists, we adapt to patient expectations of being able to engage directly with us to discuss their procedures and findings; the era of radiologists being “the doctors’ doctor”, remote from direct patient contact, is consigned to the past. Our work practices are adapted to increasing direct patient engagement, whether face to face or through our digital reports, which may be enhanced by tools to improve lay communication via patient portals [24, 25]; meeting this growing expectation will enhance the lives of both patients and radiologists, and add significant value to the services we deliver [8–10, 15, 18].

Our communication responsibilities extend also to educating our referring colleagues, patients, the general public and policy-makers about the inherently uncertain nature of much of what we do [26]. We understand the imperfection of our tools (and sometimes of our use of them), but the possibility that a radiologist may not make a correct diagnosis, or may not accurately understand or interpret abnormal findings, is often judged by members of the public or commentators to represent negligence or poor performance. As Osler wrote in 1904, “Errors in judgement must occur in the practice of an art which consists largely of balancing probabilities” [27]. Public education, led by radiologists, is required to explain the unavoidable nature of “radiological error” and the efforts we make to minimise it, while emphasising the great benefit of careful radiological practice to patients and society [28].

## The radiologist as innovator and scientist

We radiologists already contribute heavily to innovation through research; it is an unusual new treatment or drug that does not involve imaging evaluation in clinical trials to determine its value. Emerging and rapidly-developing digital and informatics-based technologies provide new opportunities for radiologists not only to contribute to but also to lead research. We enthusiastically embrace opportunities to collaborate with other stakeholders, including industry partners, in research funded by the EU and other sources, in AI, development of non-invasive cancer screening methods, clinical outcome evaluation of image-guided surgery, radiotherapy and interventional radiology technology, among other topics [29]. Modern diagnostic technologies generate promising biomarkers, not only derived from imaging, but also from genomics, tissue and blood analysis. Emerging AI applications offer the potential to integrate these biomarkers to develop accurate prediction models of outcome that will boost personalised medicine. This type of integrated diagnostics is an exciting and fast developing new field of research on the one hand that we as diagnosticians will have the opportunity to conduct and lead as well as on the other hand for the routine setting aiming at bringing all diagnostic specialties together with the goal of a collaborative report, to get even more information out of the combination of biomarkers in the future aided by AI [30]. Conduct of research by interested individuals must be funded and supported; focusing all resourcing on delivery of services means service delivery will never change, improve or advance. As with any scientific discipline, research in radiology (and all medical specialties) is intrinsic to maintaining and improving standards. We have a duty to mentor and support those among us who wish to pursue an academic career in radiology [31, 32], and this is ultimately for the benefit of all. Aside from our medical knowledge and skills, we can also bring a vital ethical perspective to research, combining our protective role with that of the innovator. This will be of immense importance in the integration of AI in medical practice [33].

## The radiologist as teacher

The Hippocratic oath, the traditional basis for the tenets of practice adopted by new medical graduates, commits us to teach our successors [34]. All doctors have a duty to devote time and effort into passing on our knowledge; this function must be provided for in our work and must be supported with time and resources. We must embrace every opportunity to teach, passing on knowledge to medical students, radiology trainees and colleagues in other specialties and other professions. The future of radiology relies upon the quality of next generation’s radiologists, and on our ability to engage and work with other specialties. Introducing radiology to medical students, demonstrating with enthusiasm the importance of our profession and stressing the role in modern medicine of the radiologist as diagnostician, interventionalist and innovative scientist is of utmost importance to attract motivated future doctors. The ESR has developed, and continuously updates, training curricula with guidelines and recommendations on undergraduate teaching of radiology, education during specialty training and subspecialisation [35].

Growing subspecialisation in radiology is a desirable development, allowing us to harness deeper knowledge and experience in focused aspects of our specialty in the service of better care. This is increasingly possible, even in smaller departments, due to the practical benefits of being able to work within wider radiology networks, with increasing digitisation of healthcare records [36]. Subspecialisation is desired by radiologists [18], beneficial to patients and referrers and should be supported by resourcing of relevant educational opportunities.

Equally, we must accept that, with time, we will be surpassed in knowledge, ability and understanding of new technologies by younger, sharper colleagues. We must pass on what knowledge we can and move aside with grace to allow others to shine when our time has passed [37].

## Conclusion

The work of the modern radiologist is complex and multi-faceted. Many non-radiologists view us as providers of imaging reports, somewhat removed from direct patient care and interaction. There is some truth to this characterisation, and this is a major aspect of what we do. However, we must ourselves recognise (and promote recognition by others) our centrality in patient care, and important aspects of our responsibilities that lie outside direct provision of image interpretation. We should take every opportunity to highlight to others the breadth of our contributions, and should devote time, attention and resources to those aspects of our role that lie outside traditional interpretive tasks. Only by refusing to be pigeon-holed as single-task automatons will our key role in patient care be maintained and developed.

## Data Availability

All relevant data is included in this manuscript.

## Abbreviations

CT: Computed tomography
ESR: European Society of Radiology
PACS: Picture archiving and communication system
